# Widespread dissemination of class 1 integron components in soils and related ecosystems as revealed by cultivation-independent analysis

**DOI:** 10.3389/fmicb.2013.00420

**Published:** 2014-01-17

**Authors:** Sven Jechalke, Susanne Schreiter, Birgit Wolters, Simone Dealtry, Holger Heuer, Kornelia Smalla

**Affiliations:** ^1^Institute for Epidemiology and Pathogen Diagnostics, Julius Kühn-Institut, Federal Research Centre for Cultivated Plants (JKI)Braunschweig, Germany; ^2^Institute of Environmental and Sustainable Chemistry, Technische Universität BraunschweigBraunschweig, Germany

**Keywords:** *intI1*, *sul1*, *qacEΔ*1, *qacE*, IncP-1 *korB*, digestates, manured soil and rhizosphere, biofilter

## Abstract

Class 1 integrons contribute to the emerging problem of antibiotic resistance in human medicine by acquisition, exchange, and expression of resistance genes embedded within gene cassettes. Besides the clinical setting they were recently reported from environmental habitats and often located on plasmids and transposons, facilitating their transfer and spread within bacterial communities. In this study we aimed to provide insights into the occurrence of genes typically associated with the class 1 integrons in previously not studied environments with or without human impact and their association with IncP-1 plasmids. Total community DNA was extracted from manure-treated and untreated soils, lettuce and potato rhizosphere, digestates, and an on-farm biopurification system and screened by PCR with subsequent Southern blot hybridization for the presence of the class 1 integrase gene *intI1* as well as *qacE* and *qacEΔ 1* resistance genes. The results revealed a widespread dissemination of class 1 integrons in the environments analyzed, mainly related to the presence of *qacEΔ 1* genes. All 28 IncP-1ε plasmids carrying class 1 integrons, which were captured exogenously in a recent study from piggery manure and soils treated with manure, carried *qacEΔ 1* genes. Based on the strong hybridization signals in the rhizosphere of lettuce compared to the potato rhizosphere, the abundances of *intI1*, *qacE/qacEΔ 1*, and *sul1* genes were quantified relative to the 16S rRNA gene abundance by real-time PCR in the rhizosphere of lettuce planted in three different soils and in the corresponding bulk soil. A significant enrichment of *intI1* and *qacE*/*qacEΔ 1* genes was confirmed in the rhizosphere of lettuce compared to bulk soil. Additionally, the relative abundance of *korB* genes specific for IncP-1 plasmids was enriched in the rhizosphere and correlated to the *intI1* gene abundance indicating that IncP-1 plasmids might have contributed to the spread of class 1 integrons in the analyzed soils.

## Introduction

Class 1 integrons are bacterial genetic elements that are widely distributed in the clinical setting as well as in the environment, where they are able to acquire, exchange, and express genes embedded in gene cassettes (Mazel, 2006; Gillings et al., 2008; Heuer and Smalla, 2012; Jaglic and Cervinkova, 2012; Stalder et al., 2012; Zhao et al., 2012; Stalder et al., 2013). These gene cassettes can contain resistance genes for almost all antibiotic families including beta-lactams, aminoglycosides, trimethoprim, chloramphenicol, fosfomycin, macrolides, lincosamides, rifampicin, and quinolones (Stalder et al., 2012). Furthermore, class 1 integrons are often located on plasmids, transposons, and flanked by insertion sequences which facilitate their transfer and spread within bacterial communities and to bacterial pathogens, which contributes to the worldwide crisis in the management of bacterial infections (Gillings et al., 2008).

Besides the selection and dissemination of class 1 integrons by antibiotics, selective pressure by heavy metals or quaternary ammonium compounds (QACs) has also been shown to be a factor involved in their dissemination since *qac* genes encoding resistance determinants to QACs are commonly found on class 1 integrons (Stalder et al., 2012). QACs are biocides which are widely distributed and applied in hospitals, industry, and cosmetics (Buffet-Bataillon et al., 2012). In the food processing industry QACs are by far the most commonly used disinfectant due to their biocidal performance as well as their non-tainting, non-toxic, and non-corrosive properties (Holah et al., 2002). Their antimicrobial activity is mainly based on interactions with phospholipids and proteins of bacterial membranes leading to the disruption of membrane integrity and leakage of cellular content (Gilbert and Moore, 2005; Ioannou et al., 2007). Resistance against QACs is mediated by *qac* genes encoding proteins involved in efflux-based multidrug pumps with relatively low specificity. Different *qac* genes have been described so far (*qacA*, *qacB*, *qacC*, *qacD*, *qacE*, *qacF*, *qacG*, *qacH*, *qacI*, *qacJ*, *qacK*, and *qacZ*), which partly share a high homology (Partridge et al., 2009; Jaglic and Cervinkova, 2012).

In the recent time there is growing concern about a possible cross-resistance between antibiotics and QACs in environmental and especially in food-associated bacteria (Morente et al., 2013). The *qacE* gene and its attenuated variant *qacEΔ 1* are widely spread in Gram-negative bacteria, which is mainly due to the high prevalence of class 1 integrons associated with *qacEΔ 1*, but may occur in Gram-positive cocci as well (Jaglic and Cervinkova, 2012). It was demonstrated for a reed bed system that QAC selection was linked to an increase in class 1 integron incidence in bacterial isolates and therefore has the potential to co-select for antibiotic resistance genes (Gaze et al., 2005). Furthermore, it was shown that the prevalence of class 1 integrons and *qac* genes in the environment exposed to detergents and/or antibiotic residues was increased (Gaze et al., 2011).

Class 1 integrons have been frequently found on IncP-1 plasmids, which are broad host range plasmids with a wide distribution in the environment (Popowska and Krawczyk-Balska, 2013). Recently, plasmids of the IncP-1ε subgroup were detected in manure and arable soil and the isolation and characterization of 50 IncP-1ε plasmids revealed that all carried class 1 integrons with highly varying sizes of the gene cassette region and *sul1* genes conferring resistance against sulfonamides, indicating their important role as vectors for horizontal transfer of antibiotic resistance in agricultural systems (Heuer et al., 2012). However, the knowledge on the distribution of *qacE* and *qacEΔ 1* genes on IncP-1ε plasmids and their role in the distribution of QAC resistance and potential co-selection of antibiotic resistance in the environment was poor.

In this study, we aimed to provide insights into the occurrence of sequences typically associated with the clinically relevant class 1 integrons in different and previously not analyzed environments to assess the potential for a co-selection of antibiotic resistance and associated risks for human health. Therefore, total community DNA from different soils, digestates, and an on-farm biopurification system was extracted and screened by PCR with subsequent Southern blot hybridization for the presence and abundance of the class 1 integrase gene *intI1* as well as *qacE* and *qacEΔ 1* resistance genes. Additionally, quantitative real-time PCR (qPCR) was used to compare the abundance of class 1 integron associated genes *intI1*, *qacE*/*qacEΔ 1*, and *sul1* with the abundance of *korB* and *trfAε* genes specific for IncP-1 and IncP-1ε plasmids, respectively.

## Materials and methods

### Origin of environmental samples and captured plasmids

Bulk soil and rhizosphere from lettuce and potato were sampled from field plots in Grossbeeren, south of Berlin, Germany, described by Rühlmann (2006), which had received mineral fertilizer only for more than 10 years. The three soil types were characterized as Arenic-Luvisol with less silty sand and 5.5% clay (diluvial sand, DS), Gleyic-Fluvisol with heavy sandy loam and 27.5% clay (alluvial loam, AL), and Luvic-Phaeozem with medium clayey silt and 17.2% clay (loess loam, LL) (Rühlmann and Ruppel, 2005).

In addition, arable soil samples amended with manure containing sulfadiazine or difloxacin or no antibiotics were taken from a field plot near Jülich, Germany, as described previously (Jechalke et al., 2013b,c).

Samples from a pesticide-degrading biofilter operated on a farm near Kortrijk, Belgium, were collected as described by Jechalke et al. (2013a). The samples were collected three times over a season before start-up (March), during processing (July), and after closedown (September).

Digestates were sampled from four different biogas plants (BGP) in Germany. From each BGP 14 ml digestate was centrifuged for 10 min (3,100 × *g*) and the pellet was homogenized.

The 28 IncP-1ε plasmids further characterized here originated from a study by Heuer and colleagues and were exogenously captured in *Escherichia coli* CV601gfp from bacterial communities of manure and manured soil (Heuer et al., 2012).

### Extraction of total community DNA

Total community (TC-) DNA was extracted from 0.5 g of soil, biofilter sample, or 0.1 g homogenized digestate pellet using the FastDNA® Spin Kit for soil (MP Biomedicals, Heidelberg, Germany). The extracted DNA from soil and biofilter samples was purified by the GeneClean® Spin Kit (MP Biomedicals), following the instructions of the manufacturer. The DNA from the digestates was diluted 1:5 in 10 mM Tris/HCl (pH 8.0) with 1 mM EDTA.

To obtain a rhizosphere pellet from the lettuce and potato plants of the Grossbeeren field site, first the loosely attached soil from three plants per replicate was removed by shaking and subsequent root washing in sterile water. Afterwards 5 g roots were suspended in 15 ml sterile 0.3% NaCl three times and treated in a Stomacher 400 Circulator (Seward, Worthing, West Sussex, UK) for 30 s at high speed. The supernatants were centrifuged (2 min, 500 × *g*) and the pellet was added to the root sample together with 15 ml sterile 0.3% NaCl for the second and third Stomacher treatment. The three supernatants were combined and centrifuged at 10,000 × *g* for 30 min to obtain the microbial pellet. The pellet was resuspended and transferred to a 2 ml reaction tube and centrifuged at 14,000 × *g* for 20 min. The resulting pellets were used for the TC-DNA extraction as described above.

### PCR with subsequent southern blot hybridization

The occurrence of *intI1*, *qacE*, and *qacEΔ 1* genes in TC-DNA was analyzed by PCR amplification and subsequent Southern blot hybridization. Primers used for the amplification of the *intI1* gene (IntI1F, IntI1R) were described by Moura et al. (2007). Primers for the amplification of *qacE* (F1, R2) and *qacEΔ 1* (qacEΔ 1 F, qacEΔ 1 B) were used as described previously, but the *qacE* primer R2 was modified (TTAGTGGGCACTTGCTTTGGAAAG) to increase its specificity (Sandvang et al., 1997; Kazama et al., 1998). Digoxigenin labeled probes were generated from PCR products as described by Jechalke et al. (2013b) using plasmids R751 and pB10 as templates for *qacE* and *qacEΔ 1*, respectively, and plasmids 1–23, 3–408, 3–414, and pKJK5 for *intI1*. Southern blotting and hybridization of PCR products were done as described by Sambrook et al. (1989). Ten μl PCR products were run on 1% agarose gels for *intI1*, *qacE*, and *qacEΔ 1.* The gels were Southern blotted to a Hybond-N membrane (GE Healthcare Limited, Amersham, UK) using standard protocols (Sambrook et al., 1989). It has to be mentioned that in case the sample only contains the *qacE* gene, the detection of *qacEΔ 1* would give a positive result, too, since the primers for *qacEΔ 1* are complementary to a region also present in the *qacE* gene.

### Quantification of target genes

The class 1 integron integrase gene *intI1* was quantified by quantitative real-time PCR 5′-nuclease assays in a CFX96 real-time PCR detection system (Bio-Rad, Hercules, CA) as described previously (Barraud et al., 2010). The quaternary ammonium compound resistance genes *qacE* and the *qacEΔ 1* variant were quantified with the primers qacEallF (CGCATTTTATTTTCTTTCTCTGGTT) and qacEallR (CCCGACCAGACTGCATAAGC) and the probe qacEallP (FAM-TGAAATCCATCCCTGTCGGTGT-TAMRA), developed in the present study, with 10 min at 95°C and 40 cycles of 30 s at 95°C and 60 s at 60°C. Standard dilutions were generated from a cloned 226 bp PCR product of the plasmid pKJK5 using primers (qacEΔ 1 F, qacEΔ 1 B) described by Sandvang et al. (1997). To assess the contribution of IncP-1 plasmids and in particular of the IncP-1ε subgroup, the abundance of *korB* genes specific for IncP-1 plasmids of all known subgroups and the abundance of *trfA* genes specific for the IncP-1ε subgroup were determined relative to 16S rRNA gene copies (*rrn*) as described by Heuer et al. (2012) and Jechalke et al. (2013a).

The 16S rRNA genes were quantified using the primers BACT1369F and PROK1492R and the probe TM1389F (Suzuki et al., 2000). Differences in bacterial DNA and amplification efficiency between samples were adjusted by dividing the target numbers of the respective genes by the *rrn* gene copy numbers and the log transformation of the results (relative abundance).

The correlation between the relative abundance of *intI1* and the class 1 associated genes *qacE/qacEΔ 1* and *sul1* as well as the relative abundance of *korB* was tested by linear regression analysis (*p* < 0.05; SAS 9.3; SAS Institute Inc., Cary, NC).

## Results

### Gene detection and quantification in environmental samples

**Figure 1:**
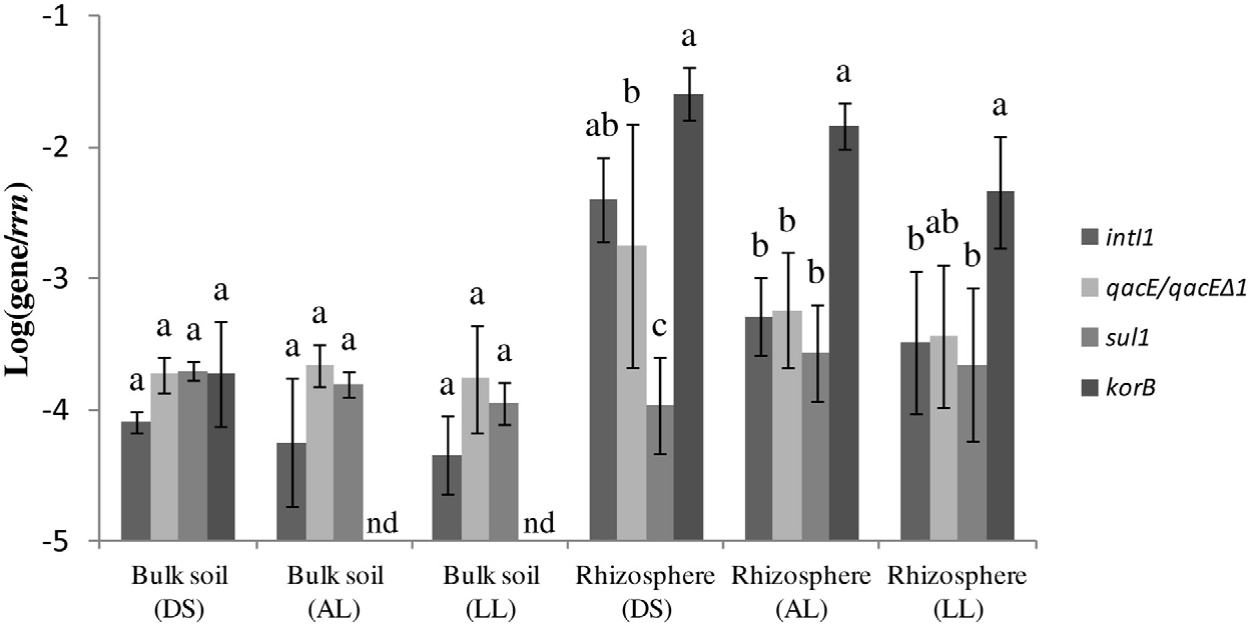
**Abundance of class 1 integron integrase gene *intI1*, quaternary ammonium compound resistance genes *qacE/qacE Δ 1*, sulfonamide resistance gene *sul1*, and *korB* genes related to IncP-1 plasmids relative to 16S rRNA gene copies (*rrn*)**. Results are shown for bulk soil and rhizosphere of lettuce for the three soil types: alluvial loam (AL), diluvial sand (DS), and loess loam (LL). Error bars represent standard deviations of *n* = 4 replicates or of *n* = 3 replicates for bulk soil (DS). Values below detection limit (−4.6) of the qPCR assay for *korB* are indicated (nd). Pairwise comparisons were made for each soil type of bulk soil and rhizosphere separately. Differing letters indicate significant differences between relative abundances of target genes (Tukey test, *p* < 0.05).

PCR with subsequent Southern blot hybridization revealed that all tested TC-DNAs extracted from manure-treated soil, lettuce and potato rhizosphere, digestates, and biofilter samples contained *intI1* genes and the *qacEΔ 1* gene variant but at very different abundances as indicated by the hybridization signal intensity (Table 1, Figures S1–S3). The *qacE* gene was found in a lower frequency and was observed only in the biofilter samples (Table 1), in the rhizosphere of lettuce grown in DS soil, and in one replicate of potato rhizosphere from LL soil (Table 1). As the hybridization signals were strongly increased in the TC-DNA from lettuce rhizosphere compared to potato rhizosphere, PCR amplifications from lettuce rhizosphere and the corresponding bulk soil were repeated and the hybridization signals indicated an enrichment of populations carrying *intI1* and *qacEΔ 1* in the rhizosphere of lettuce grown in all three soil types (Figures S4, S5). This result was confirmed by quantifying the class 1 integron integrase genes *intI1* and quaternary ammonium compound resistance genes *qacE*/*qacEΔ 1* by qPCR. In accordance with the results from Southern blot hybridization, all samples contained *intI1* and *qacE*/*qacEΔ 1* genes (Figure 1). The abundances of *intI1* and *qacE*/*qacEΔ 1* relative to 16S rRNA genes were significantly higher in the rhizosphere than in bulk soil (*t*-test, *p* < 0.05). The relative abundance of *sul1* was not significantly different between bulk soil and rhizosphere (*t*-test, *p* < 0.05). The relative abundance of *intI1*, *qacE*/*qacEΔ 1*, and *sul1* was not significantly different among the different soil types in bulk soil or rhizosphere, only in the rhizosphere of DS soil the relative abundance of *sul1* was lower (Tukey test, *p* < 0.05). Linear regression analysis revealed a significant correlation between the relative abundances of *intI1* and *qacE*/*qacEΔ 1* (*p* = 0.0003, *R*^2^ = 0.46) but not between *intI1* and *sul1* (*p* = 0.63, *R*^2^ = 0.01).

**Table 1:** **Detection of class 1 integron integrase genes (*intI1*) and quaternary ammonium compound resistance genes *qacE* and *qacEΔ1* by PCR and subsequent Southern blot hybridization**.

**Sample type**		***IntI1***	***qacE***	***qacE Δ1*^a^**
Biogas plant	Digestate (BGA1)	+++	−	++
	Digestate (BGA2)	+++	−	++
	Digestate (BGA7)	+++	−	++
	Digestate (BGA9)	+++	−	++
Bulk soil Merzenhausen	Manure +difloxacin	++	−	++
	Manure	++	−	++
	Manure +sulfadiazine	++	−	++
	Manure	++	−	++
Biofilter Kortrijk	Before startup (March)	+++	+	++
	During processing (July)	+++	++	++
	After closedown (September)	+++	++	++
Lettuce rhizosphere^b^	DS soil	+++	+	++
	AL soil	+++	−	++
	LL soil	+++	−	++
Potato rhizosphere^b^	DS soil (1)	+++	−	++
	DS soil (2)	++	−	+
	DS soil (3)	+	−	+
	DS soil (4)	++	−	+
	AL soil (1)	+	−	+
	AL soil (2)	+	−	+
	AL soil (3)	++	−	+
	AL soil (4)	+	−	+
	LL soil (1)	++	−	+
	LL soil (2)	+	−	+
	LL soil (3)	+	+	+
	LL soil (4)	+	−	+

Total community DNA used as template was extracted from digestates of four different biogas plants, from soil of an agricultural field located near Merzenhausen, Germany, from a biofilter near Kortrijk, Belgium, for the remediation of pesticide contaminated water, as well as from lettuce and potato rhizosphere from field plots located in Grossbeeren, Germany. Different intensities in fluorescence are indicated (+, ++, +++).

a In case the sample only contains qacE the detection of qacEΔ 1 would also give a positive result since the primers and probe for qacEΔ 1 are complementary to a region also present in the qacE gene.

^b^Diluvial sand (DS), alluvial loam (AL), and loess loam (LL).

To assess a possible link between IncP-1 plasmids and the prevalence of class 1 integrons and associated genes, the abundance of *korB* genes specific for IncP-1 plasmids of all known subgroups was determined relative to 16S rRNA genes. In AL and LL bulk soil, the relative abundance of *korB* was below the detection limit (−4.6) of the qPCR assay. In the DS bulk soil the relative abundance of *korB* was not significantly different from the abundance of *intI1*, *qacE*/*qacEΔ 1*, and *sul1* (Tukey test, *p* < 0.05). In the AL rhizosphere, the relative abundance of *korB* was significantly higher than the relative abundance of *intI1*, *qacE*/*qacEΔ 1*, and *sul1* (Figure 1)*.* In the rhizosphere of LL soil, the relative abundance of *korB* was higher than the relative abundances of *intI1* and *sul1* but not significantly higher than the relative abundance of *qacE*/*qacEΔ 1*. In the rhizosphere of DS soil, the relative abundance of *korB* was higher than the relative abundances of *qacE*/*qacEΔ 1* and *sul1* but not significantly higher than *intI1* of class 1 integrons. Overall, in the rhizosphere the relative abundance of the *korB* gene was higher than in DS bulk soil (Figure 1, *t*-test, *p* < 0.05). Linear regression analysis revealed a significant correlation between the relative abundance of *intI1* and *korB* (*p* = 0.0012, *R*^2^ = 0.57). Additionally, the relative abundance of the *trfA* genes specific for the IncP-1ε subgroup was determined. Only in two replicates of LL rhizosphere a value clearly above the detection limit (−4.6) of the qPCR assay could be detected (data not shown).

### Detection of *qacE* and *qacEΔ 1* on exogenously isolated Incp-1ε plasmids

IncP-1ε plasmids which were previously captured by exogenous plasmid isolation from different environments were tested for the presence of class 1 integron integrase genes *intI1* and quaternary ammonium compound resistance genes *qacE* and *qacEΔ 1* by PCR and subsequent Southern blot hybridization. All IncP-1ε plasmids carried *intI1* and *qacEΔ 1* while *qacE* was not detected (Table 2). No difference in the presence of *intI1* and *qacE*/*qacEΔ 1* was observed between plasmids captured from different environmental compartments such as soil and manure and between the experiments.

**Table 2:** **IncP-1ε plasmids captured by exogenous plasmid isolation from different environments (Heuer et al., 2012) tested for the presence of class 1 integron integrase genes (*intI1*) and quaternary ammonium compound resistance genes *qacE* and *qacE Δ1* by PCR and subsequent Southern blot hybridization**.

**Plasmid**	**Source**	***IntI1***	***qacE***	***qacEΔ1***
2−S2	Manure	+	−	+
2−S5	Manure	+	−	+
3−S1	Manure	+	−	+
6−S1	Manure	+	−	+
9−T4	Manure	+	−	+
1–83	Soil microcosm	+	−	+
1–91	Soil microcosm	+	−	+
1–111	Soil microcosm	+	−	+
1–115	Soil microcosm	+	−	+
1–127	Soil microcosm	+	−	+
1–131	Soil microcosm	+	−	+
1–135	Soil microcosm	+	−	+
1–146	Soil microcosm	+	−	+
1–163	Soil microcosm	+	−	+
1–167	Soil microcosm	+	−	+
2–238	Soil microcosm	+	−	+
3–407	Soil microcosm	+	−	+
3–409	Soil microcosm	+	−	+
3–420	Soil microcosm	+	−	+
3–427	Soil microcosm	+	−	+
3–428	Soil microcosm	+	−	+
C 131	Soil mesocosm	+	−	+
253	Field soil	+	−	+
260	Field soil	+	−	+
263	Field soil	+	−	+
267	Field soil	+	−	+
268	Field soil	+	−	+
269	Field soil	+	−	+

## Discussion

The class 1 integron integrase gene *intI1* and quaternary ammonium compound resistance gene *qacEΔ 1* were detected by PCR-Southern blot hybridization in all samples of manure-treated arable bulk soil as well as in lettuce and potato rhizosphere, digestates of biogas plants, and biofilter material (Table 1), although at very different abundance. Consequently, the present study expands the environmental settings in which class 1 integrons were detected and the findings are in line with the previously reported dissemination of these genes in natural environments and environments with anthropogenic impact (Gillings et al., 2008; Gaze et al., 2011). In contrast to *qacEΔ 1*, the gene *qacE* was only detected by PCR-Southern blot hybridization in the samples from a biofilter used to degrade pesticides as well as in one replicate of potato rhizosphere of LL soil and in lettuce rhizosphere of DS soil, indicating a lower distribution of this gene variant in the environment. A low abundance of *qacE* was also observed by Chuanchuen et al. (2007) who could detect *qacEΔ 1* but not *qacE* genes in *Salmonella enterica* isolates from poultry and swine as well as by Farkas et al. (2013) who could detect *qacEΔ 1* but not *qacE* genes in biofilm samples collected at a drinking water treatment plant facility. Furthermore, in clinical isolates of Gram-negative bacteria *qacEΔ 1* was found in 10% of the isolates (*n* = 103) while *qacE* was only found in one *P. aeruginosa* strain (Kücken et al., 2000). Kazama and colleagues studied the presence of *qacE* and *qacEΔ 1* in clinical and environmental isolates from Japan. While *qacEΔ 1* was detected frequently in isolates from the clinic and the environment, *qacE* could be only detected in clinical isolates of *P. aeruginosa* (Kazama et al., 1998).

Interestingly, the initial PCR-Southern blot hybridization based screening indicated that the *intI1* and the *qacEΔ 1* genes were strongly enriched in the lettuce rhizosphere compared to potato rhizosphere samples. This finding is in accordance with the plant species dependent bacterial community composition in the rhizosphere (Berg and Smalla, 2009). Quantitative real-time PCR was performed to confirm the enrichment of class 1 integrons and quaternary ammonium resistance genes *qacE* and *qacEΔ 1* in the rhizosphere of lettuce. Furthermore, *sul1* genes were quantified, which are typically associated with *qacEΔ 1* at the 3′ conserved segment, a structural organization also described for “clinical integrons” (Gillings et al., 2009; Stalder et al., 2012). In the rhizosphere samples in general, a significantly higher abundance of *intI1* and *qacE*/*qacEΔ 1* but not of *sul1* relative to 16S rRNA genes was observed compared to the bulk soil samples, and linear regression analysis showed a correlation between *intI1* and *qacE*/*qacEΔ 1* but no significant correlation between *intI1* and *sul1* relative abundance, which may indicate an increased abundance of integron types not following the typical structural organization of clinical integrons. However, except for DS rhizosphere, the relative abundances of *intI1*, *qacE*/*qacEΔ 1*, and *sul1* were not significantly different within one soil type, indicating that the prevalent class 1 integrons mainly consisted of the clinically related *qacEΔ 1-sul1* type. In the rhizosphere of DS soil the relative abundance of *sul1* genes was lower than of *intI1* and *qacE*/*qacEΔ 1* genes, probably due to a higher abundance of class 1 integrons with the *qacE* gene located at the 3′ conserved segment or the presence of other class 1 integron types lacking the *sul1* gene, as for example observed in a commensal population of bacteria from patients in an intensive care unit (Betteridge et al., 2011). In accordance with this, only in DS rhizosphere *qacE* was detected by PCR with subsequent Southern blot hybridization (Figure S2).

Class 1 integrons are not self-transferable elements and therefore depend on the association with mobile genetic elements such as transposons and plasmids for their dissemination among bacteria. Plasmids of the incompatibility group IncP-1 were isolated from different environments and are known to spread genes between distinct phylogenetic groups of bacteria, including genes conferring resistance against antibiotics or QACs (Schlüter et al., 2007; Heuer and Smalla, 2012; Popowska and Krawczyk-Balska, 2013). Six distinct phylogenetic clades have been described, including α, β, γ, δ, ε, and ζ, which differ in host range (Yano et al., 2013). IncP-1ε plasmids have been found in different environments such as estuarine waters (Oliveira et al., 2012), agricultural soils (Sen et al., 2011), the influent of a Danish wastewater treatment plant (Bahl et al., 2009), and they could be frequently captured from manure and manure-treated soils (Heuer et al., 2012). Recently, Heuer et al. (2012) characterized fifty IncP-1ε plasmids exogenously captured from manure and manure-treated arable soil into an *E. coli* recipient. All of them carried class 1 integrons with highly varying sizes of the gene cassette region and the *sul1* gene and therefore the authors of this study suggested that IncP-1ε plasmids might be important vectors for horizontal transfer of antibiotic resistance in agricultural systems (Heuer et al., 2012). Here, we could show that all 28 plasmids selected from these IncP-1ε plasmids contain additionally the *qacEΔ 1* but not the *qacE* gene which is in accordance to the widely observed 3′ conserved segment composed of *qacEΔ 1* and *sul1* (Table 2)*.* To assess the distribution and abundance of IncP-1 plasmids containing class 1 integrons in an agricultural model system, their abundance was determined by quantitative real-time PCR in the rhizosphere of lettuce and the corresponding bulk soil.

The *korB* genes specific for IncP-1 plasmids could be detected in all lettuce rhizosphere samples but only in the DS bulk soil the relative abundance of *korB* was above the detection limit of the qPCR assay (Figure 1). This is reasonable because IncP-1 plasmids are often scarce in soil (Heuer and Smalla, 2012). Additionally, DS soil has a high proportion of sand and gravel which probably exerted a selective pressure on the bacterial community by more intensive drying events and lower nutrient contents compared to the two other soils leading to a selective advantage of plasmid carrying populations with a higher genetic flexibility. In the rhizosphere samples, corresponding to the enrichment of class 1 integrons and *qacEΔ 1* resistance genes, the relative abundance of *korB* carrying populations was significantly higher than in DS bulk soil. This is in line with the consideration that the rhizosphere in general is a hot spot for bacterial activity and horizontal gene transfer (van Elsas et al., 2003; Heuer and Smalla, 2012), which seems to be triggered by exudation and root growth affecting the cell density, distribution and metabolic activity (Mølbak et al., 2007). Furthermore, IncP-1 plasmids are known to carry catabolic genes and were shown to efficiently transfer in the rhizosphere (Pukall et al., 1996; Top and Springael, 2003; Mølbak et al., 2007; Heuer et al., 2012; Król et al., 2012; Popowska and Krawczyk-Balska, 2013), which might indicate that root exudates might have selected for populations carrying catabolic plasmids. This hypothesis might be supported by a significant enrichment of *Variovorax* populations in the rhizosphere of all three soil types (data not published), a genus which is known to be able to carry catabolic plasmids of the IncP-1 group (Kim et al., 2013). Interestingly, in the DS bulk soil the relative abundance of IncP-1 plasmids was similar to the abundance of class 1 integron associated genes *intI1*, *qacE*/*qacEΔ 1*, and *sul1* (Figure 1), which indicated that class 1 integrons with typical structural organization of clinical integrons might have been associated with IncP-1 plasmids. Furthermore, linear regression analysis of all bulk and rhizosphere samples showed a significant correlation of the relative abundance of *intI1* and *korB* genes, supporting this assumption. However, in the rhizosphere samples the relative abundance of *korB* genes in the three soils was significantly higher than most of the class 1 integron components (Figure 1), indicating also a high prevalence of IncP-1 plasmids not carrying class 1 integrons. Plasmids of the IncP-1ε subgroup, which were captured from manure and manured soil were only detected in two of the four replicates of LL rhizosphere (data not shown) indicating only a minor contribution of this subgroup in the distribution of class 1 integrons in the soils which did not receive manure in the last 10 years. This is in line with the results reported by Heuer et al. (2012) who detected only a very low abundance of IncP-1ε plasmids in arable soils. However, they could show that the application of manure spiked with the antibiotic sulfadiazine increased the relative abundance of IncP-1ε plasmids, which might be related to co-selection of class 1 integrons with the *sul1* resistance gene located on these plasmids.

## Concluding remarks

In summary, this study provided insights into the occurrence of class 1 integron components in previously not studied environments such as on-farm biopurification systems, digestates, and soils treated or untreated with manure. The high prevalence of class 1 integron components in samples from manured soils, on-farm pesticide biopurification systems and digestates was not unexpected considering the use of piggery manure in all these settings. However, the detection of class 1 integron components in soils which for at least 10 years were only fertilized with minerals and the enrichment of class 1 integrons and IncP-1 plasmids carrying bacteria in the rhizosphere which most likely was a response to root exudation were striking findings of the present study. The quantification of IncP-1 plasmids revealed a potential association with class 1 integrons in bulk soil, while in the rhizosphere of lettuce the relative abundance of IncP-1 plasmids was much higher than the abundance of class 1 integrons. Plasmids of the IncP-1ε subgroup previously identified as important vectors of antibiotic resistance genes in manure and manured soil, likely had a small contribution to the presence of class 1 integrons in the investigated arable soil which did not receive manure for at least 10 years. The observed high prevalence of *intI1* and *qacEΔ 1* genes in the environment and their potential localization on broad host range plasmids may represent a constant reservoir for the spread of these genes into hospitals, food industry, or other man-made environments where QACs are used for biocidal purposes, which may lead to a co-selection of class 1 integrons and associated antibiotic resistance genes. Exogenous isolation of IncP-1 plasmids from the rhizosphere of lettuce will be required to confirm the localization of *qacEΔ 1* and the environmental factors which likely triggered the increased abundance of IncP-1 plasmid carrying bacteria in the rhizosphere of lettuce.

### Conflict of interest statement

The authors declare that the research was conducted in the absence of any commercial or financial relationships that could be construed as a potential conflict of interest.

## Supplementary material

Figure S1 **PCR amplification from total community DNA and subsequent Southern blot hybridization of class 1 integron integrase gene *intI1***.

Figure S2 **PCR amplification from total community DNA and subsequent Southern blot hybridization of quaternary ammonium compound resistance gene *qacE***.

Figure S3 **PCR amplification from total community DNA and subsequent Southern blot hybridization of quaternary ammonium compound resistance gene *qacEΔ 1***.

Figure S4 **PCR amplification from total community DNA and subsequent Southern blot hybridization of class 1 integron integrase gene *intI1***.

Figure S5 **PCR amplification from total community DNA and subsequent Southern blot hybridization of quaternary ammonium compound resistance gene *qacEΔ 1***.
