# Supplementary material for: Widespread dissemination of class 1 integron components in soils and related ecosystems as revealed by cultivation-independent analysis
Source: Front Microbiol. 2014 Jan 17;4:420. doi: 10.3389/fmicb.2013.00420 (PMC3894453; doi:10.3389/fmicb.2013.00420)
Supplement: Figure S1 — PCR amplification from total community DNA and subsequent Southern blot hybridization of class 1 integron integrase gene intI1. [file DataSheet1.PDF]

Supplemental data

Widespread dissemination of class 1 integron components in soils and related ecosystems as revealed by cultivation-independent analysis

Sven Jechalke[1], Susanne Schreiter[1], Birgit Wolters[1,2], Simone Dealtry[1], Holger Heuer[1], Kornelia Smalla[1*]
[1]Julius Kühn-Institut, Federal Research Centre for Cultivated Plants, Institute for Epidemiology and Pathogen Diagnostics, Braunschweig, Germany
[2]Technische Universität Braunschweig, Institute of Environmental and Sustainable Chemistry, Braunschweig, Germany

Figures: 5

Correspondence:
Prof. Dr. Kornelia Smalla
Julius Kühn-Institut, Institute for Epidemiology and Pathogen Diagnostics
Messeweg 11-12
38104 Braunschweig, Germany
kornelia.smalla@jki.bund.de

Figures

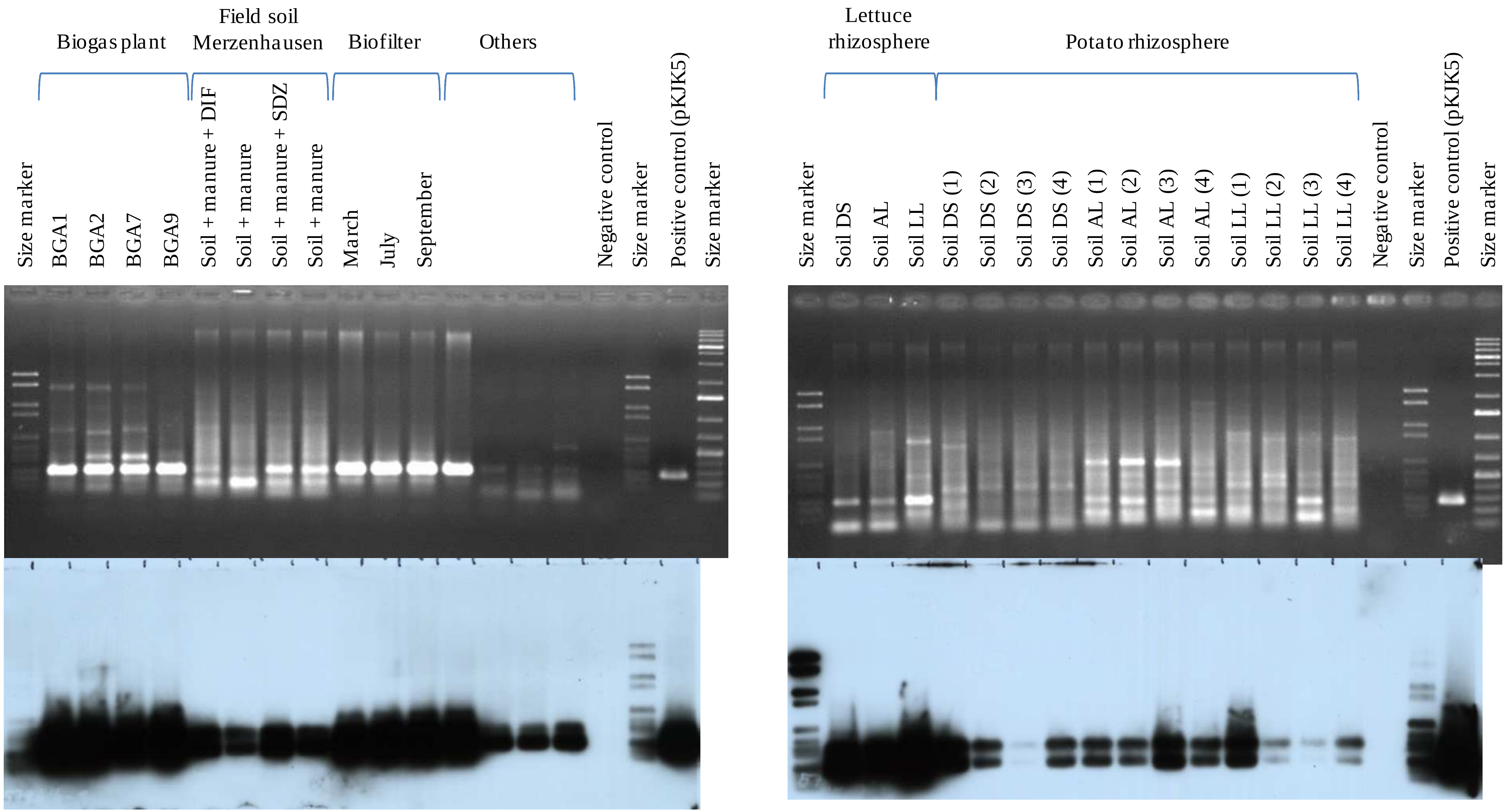

Figure S1: PCR amplification from total community DNA and subsequent Southern blot hybridization of class 1 integron integrase gene *intI1*.

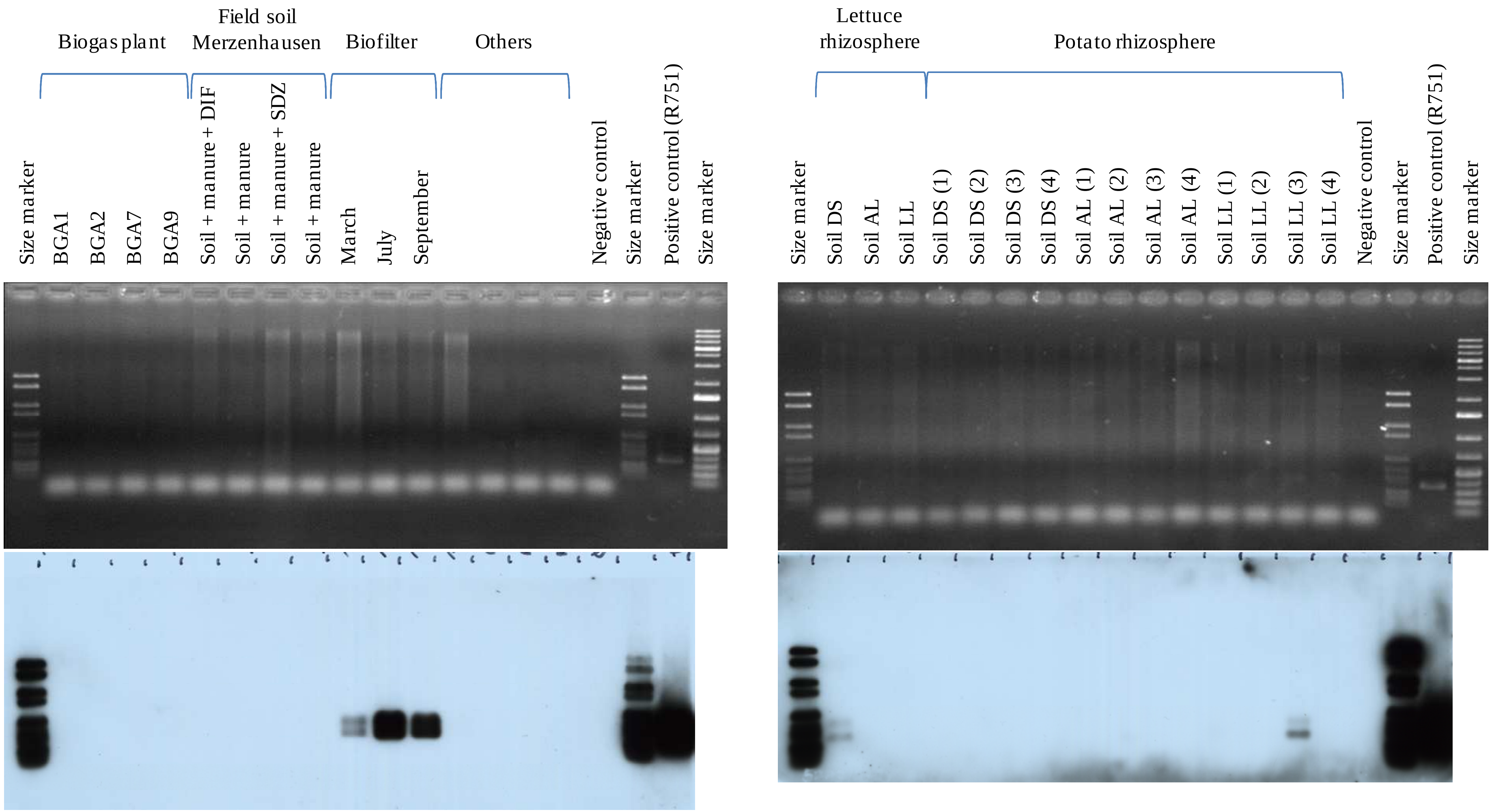

Figure S2: PCR amplification from total community DNA and subsequent Southern blot hybridization of quaternary ammonium compound resistance gene *qacE*.

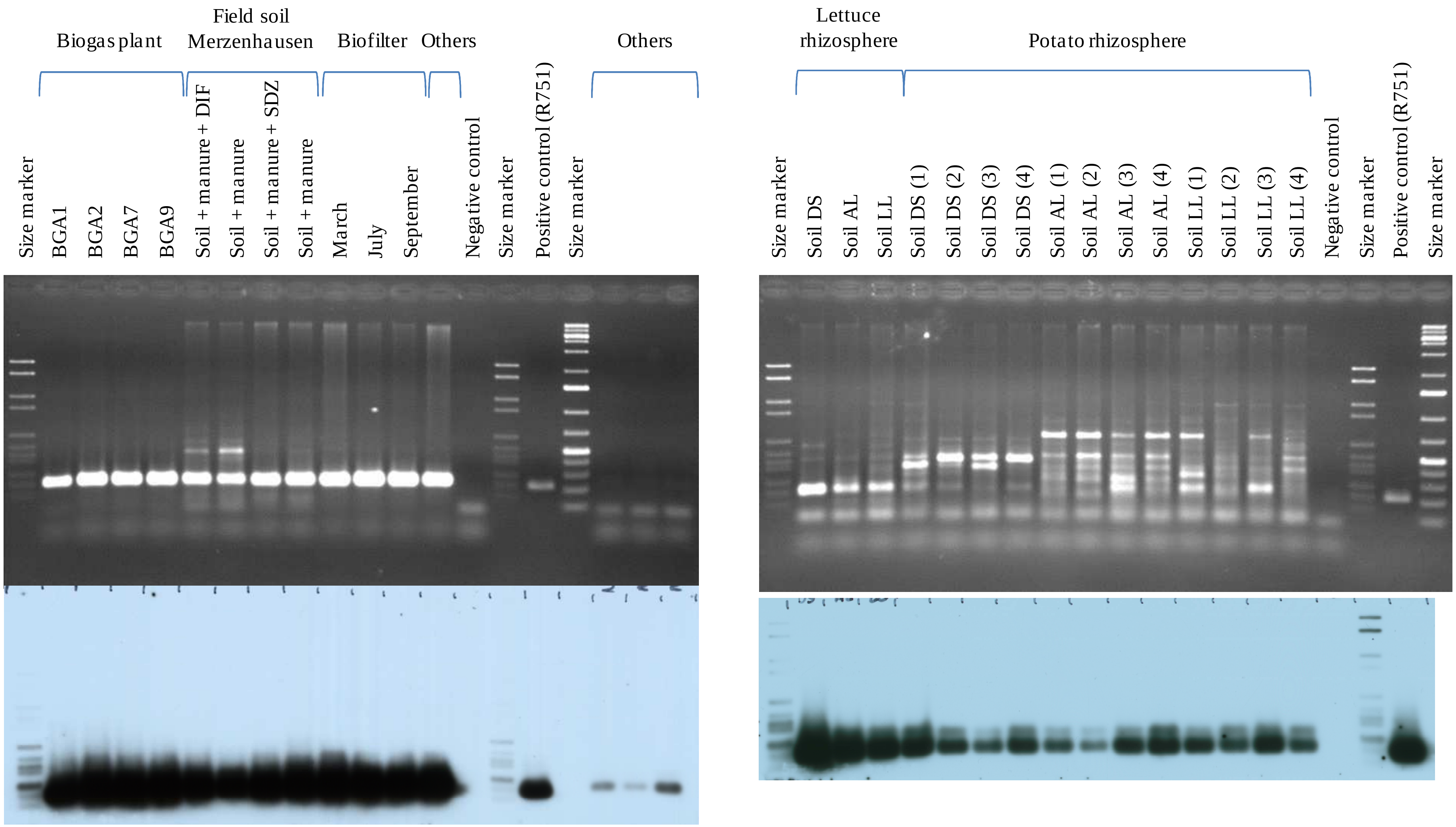

Figure S3: PCR amplification from total community DNA and subsequent Southern blot hybridization of quaternary ammonium compound resistance gene *qacEΔ1*.

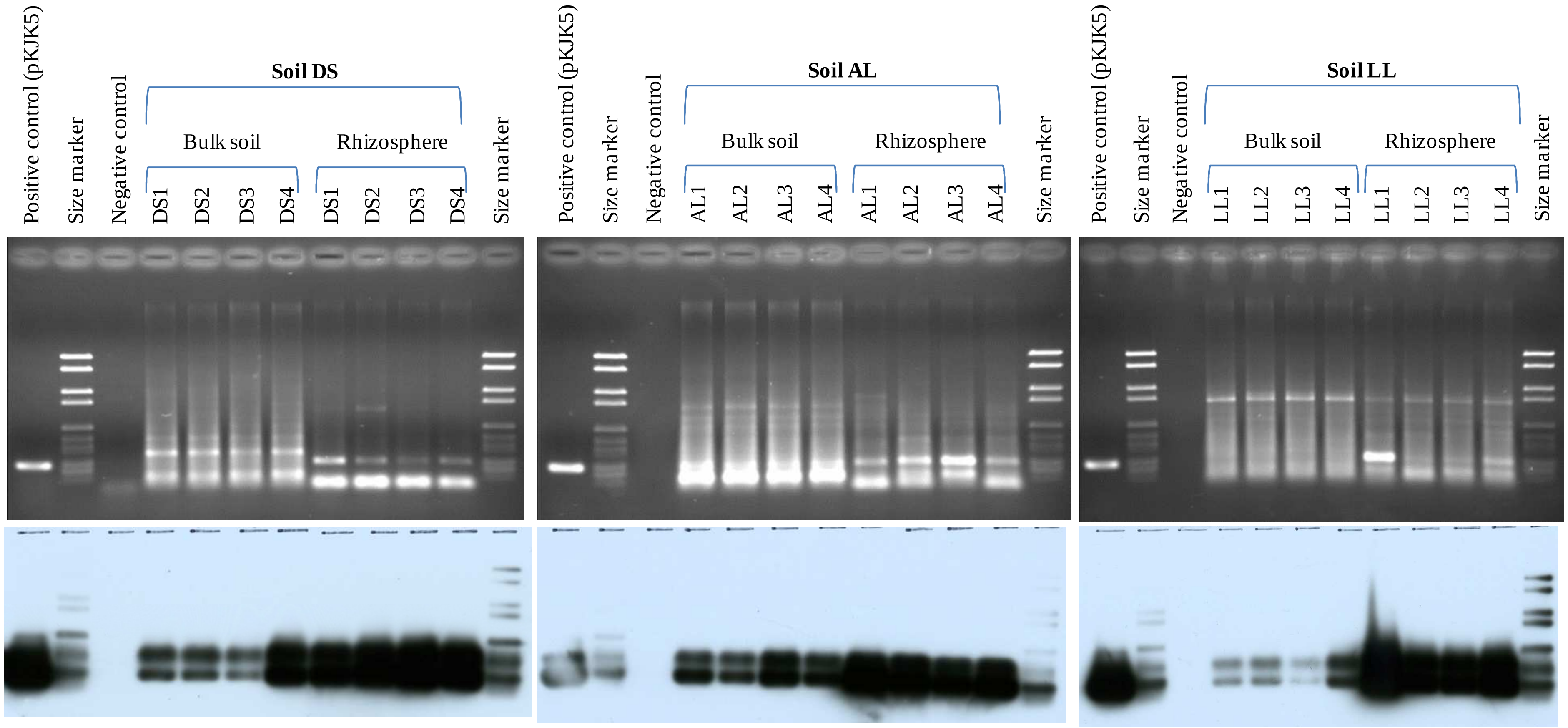

Figure S4: PCR amplification from total community DNA and subsequent Southern blot hybridization of class 1 integron integrase gene *intI1*.

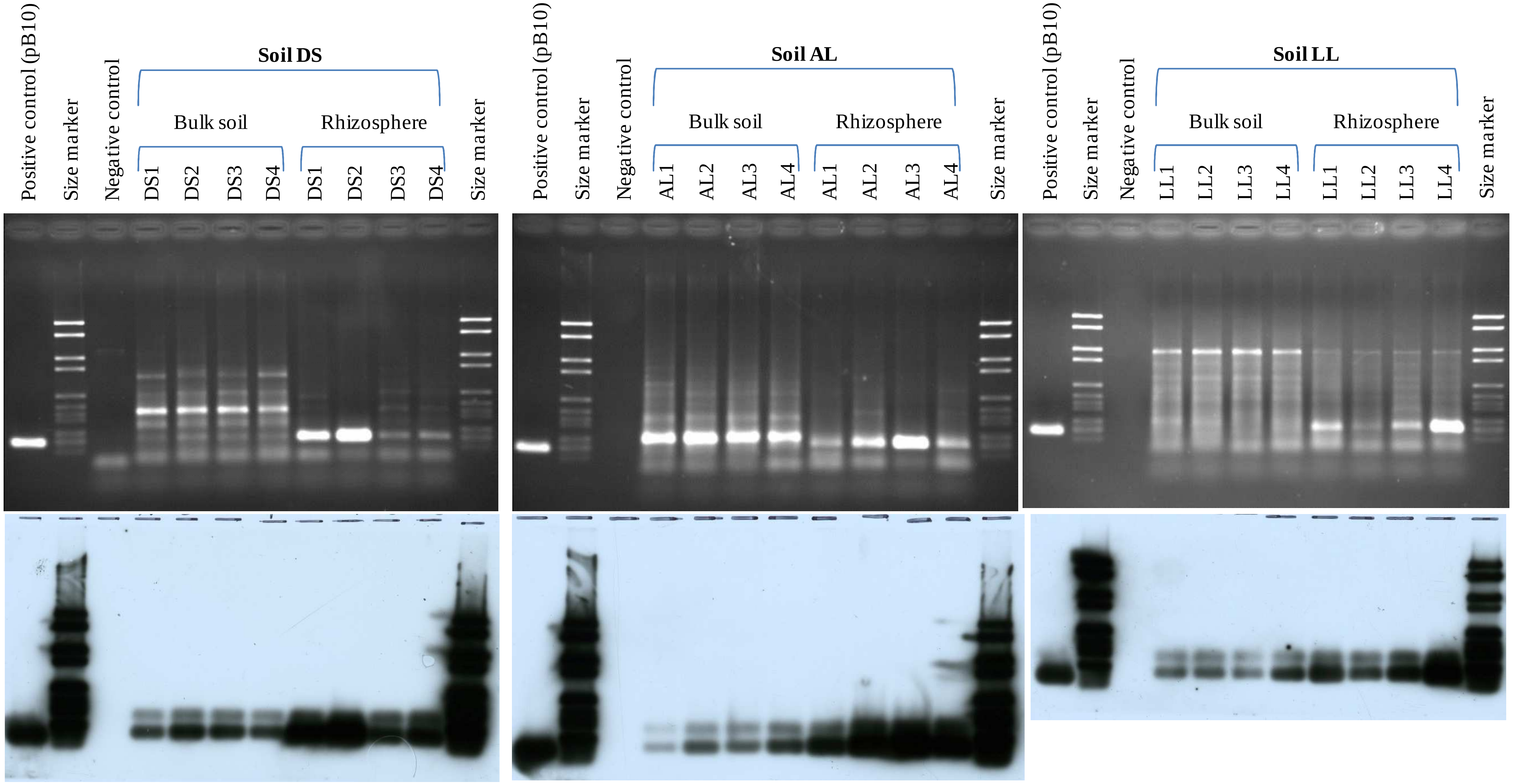

Figure S5: PCR amplification from total community DNA and subsequent Southern blot hybridization of quaternary ammonium compound resistance gene *qacEΔ1*.
